# Resolving the Paradox of Colon Cancer Through the Integration of Genetics, Immunology, and the Microbiota

**DOI:** 10.3389/fimmu.2020.600886

**Published:** 2020-12-14

**Authors:** Marine Fidelle, Satoru Yonekura, Marion Picard, Alexandria Cogdill, Antoine Hollebecque, Maria Paula Roberti, Laurence Zitvogel

**Affiliations:** ^1^ Gustave Roussy, Villejuif, France; ^2^ Institut National de la Santé Et de la Recherche Médicale (INSERM) U1015, Villejuif, France; ^3^ Equipe Labellisée—Ligue Nationale contre le Cancer, Villejuif, France; ^4^ Center of Clinical Investigations in Biotherapies of Cancer (CICBT) 1428, Villejuif, France; ^5^ Université Paris-Saclay, Gustave Roussy, Villejuif, France; ^6^ Unit Biology and Genetics of the Bacterial Cell Wall, Institut Pasteur, Paris, France; ^7^ Department of Immunology, University of Texas, MD Anderson Cancer Center, Houston, TX, United States; ^8^ Department of Genomic Medicine, University of Texas, MD Anderson Cancer Center, Houston, TX, United States; ^9^ Department of Medical Oncology, Gustave Roussy, Villejuif, France

**Keywords:** colon cancer, immunity, *Bacteroides fragilis*, *Fusobacterium nucleatum*, ileum, microbiome, immune checkpoint

## Abstract

While colorectal cancers (CRC) are paradigmatic tumors invaded by effector memory lymphocytes, the mechanisms accounting for the relative resistance of MSI negative CRC to immunogenic cell death mediated by oxaliplatin and immune checkpoint inhibitors has remained an open conundrum. Here, we propose the viewpoint where its microenvironmental contexture could be explained -at least in part- by macroenvironmental cues constituted by the complex interplay between the epithelial barrier, its microbial ecosystem, and the local immune system. Taken together this dynamic ménage-à-trois offers novel coordinated actors of the humoral and cellular immune responses actionable to restore sensitivity to immune checkpoint inhibition. Solving this paradox involves breaking tolerance to crypt stem cells by inducing the immunogenic apoptosis of ileal cells in the context of an ileal microbiome shifted towards immunogenic bacteria using cytotoxicants. This manoeuver results in the elicitation of a productive Tfh and B cell dialogue in mesenteric lymph nodes culminating in tumor-specific memory CD8^+^ T cell responses sparing the normal epithelium.

## The Paradox of Colon Cancer

### Introduction

Colorectal cancer (CRC) is the third leading malignancy worldwide and the second most common cause of cancer mortality, regardless of gender. CRC is expected to increase in incidence by 60% by 2030, posing an increasing burden on health care systems worldwide (1). The typical development of CRC takes place in aberrant crypts, evolving over time into a neoplastic precursor lesion called a “polyp.” The carcinogenic evolution is thought to occur over 10–15 years, following a traditional adenoma-carcinoma tumor progression. CRCs are often divided into two groups delineated by location: left-sided and right-sided (proximal) colon cancers. These anatomical sites help to capture the heterogeneous features of CRC in relation to key physiological landmarks (2).

**Figure 1:**
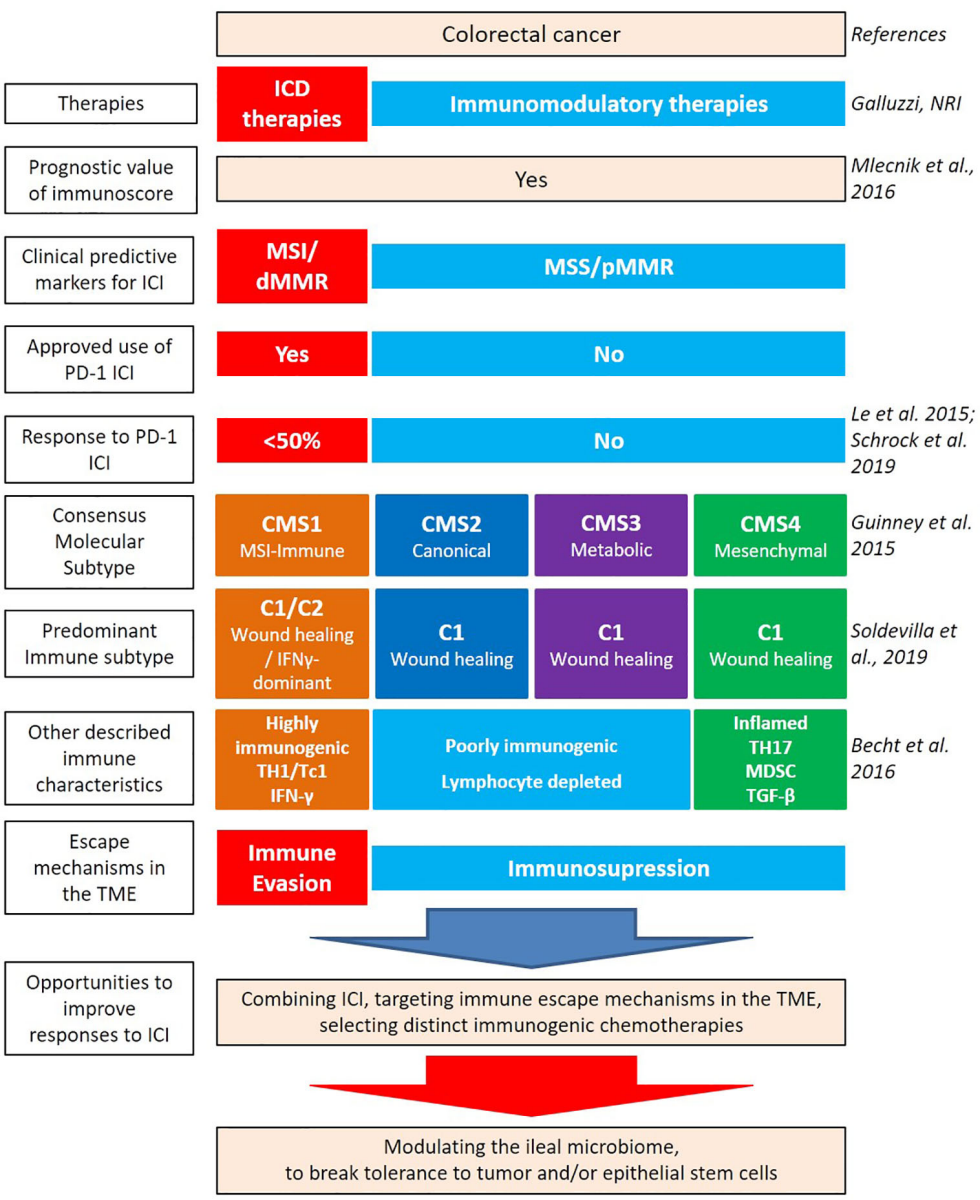
Heterogeneity of colorectal cancer (CRC) leading to new classifications. Several factors contribute to the intrinsic immunogenicity of CRC. There are several classifications based on anatomical, genetic, and immunological parameters which allow for the dissection of the intertwined relationships between these components and to predict clinical outcome. Therapies can contribute to gears of intrinsic immunogenicity, by providing antigens and adjuvants. While oxaliplatin (OXA)-based chemotherapeutic regimen and anti-EGFR Abs can be considered “immunogenic” therapeutics, paving the way to a better efficacy of immune checkpoint inhibitors (ICI), anti-VEGF Abs could rather modulate the vascularization and distinct immunosuppressive cues of the tumor microenvironment (TME) [such as myeloid-derived suppressor cell (MDSC)]. Despite this knowledge, the combination of immunogenic cell death-mediating compounds with ICI failed to ameliorate CRC patients’ prognosis, at least in MSS CRC. Integrating the ileal microbiome in this equation has the potential to break tolerance to self-antigens of the crypts, by priming Tfh and B cell responses, instrumental to control tumor progression.

CRC results from a progressive accumulation of epigenetic and genetic alterations resulting in the transformation of normal colonic mucosa to adenocarcinoma. 60%–65% of CRCs are classified as sporadic, occurring in people without a family history or genetic predisposition (3). Sporadic CRC development is often associated with numerous risk factors related to health determinants such as lifestyle, diet, smoking, and alcohol consumption (4). Key determinants such as smoking status is linked to proximal colorectal cancer (pCC) and rectal cancer development (5). While dietary habits such as overt consumption of animal fat, red and processed meat, low intake of dietary fibers, unrefined grains and vegetables promote key inflammatory pathways associated with CRC (6, 7). The current staging or classification of CRC malignancies, such as TNM (8, 9) only takes into account primary tumor size, regional lymph node involvement, and metastatic spread but fails in the ability to further delineate how best to approach therapeutic management of malignancies. Indeed, key aspects of classification, such as genetic specificities, immunological contexture, and gut dysbiosis, play a vital role in the physiopathology of CRC and should be considered in context (**Figure 1**) (10).

### Genetic and Immunological Traits of Colon Cancers

CRC arises from mutational activation of oncogenes associated with the mutational inactivation of tumor suppressor genes (11). Three non-exclusive major types of genomic instability have been described in CRC. The first type, occurring in 85% of CRC, concerns gene mutations in *APC* or other tumor suppressor genes resulting in activation of the Wnt pathway characterized by a chromosomal instability (CIN) phenotype. The second type found in 20%–30% of CRC, accounts for global genome hypermethylation coinciding with the inactivation of tumor suppressor genes, known as CpG island methylator phenotype (CIMP) (12). The last type is found in ~15% of patients who encounter the loss of DNA mismatch repair (MMR), leading to a high level of microsatellite instability (MSI-High), a hypermutable phenotype (13). The MSI-H phenotype results from either a somatic inactivation of *MLH1* MMR gene (sporadic cases, 12%) or from a germline mutation in MMR genes (*MLH1, MSH2, MSH6, PMS2*) leading to a deficient DNA mismatch repair (dMMR); such as in the case of the Lynch syndrome (3%) (14). The CIMP phenotype can lead to the MSI phenotype when hypermethylation of the *MLH1* gene promoter occurs. This particular MSI phenotype generates neoantigens accounting for their intrinsic immunogenicity (15).

**Figure 2:**
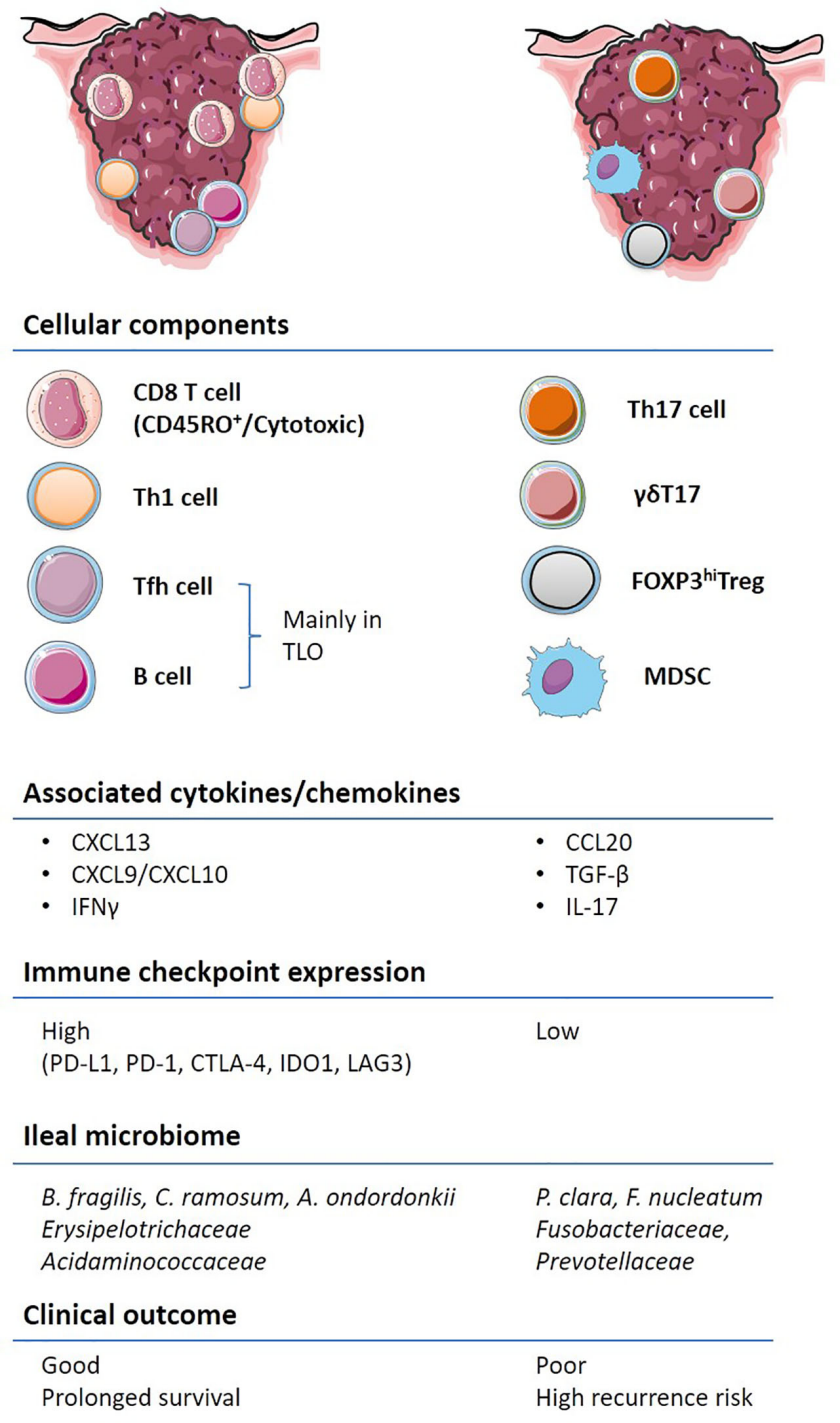
Immune contexture of primary and metastatic colorectal cancer (CRC). A non-exhaustive list of the main immune features contributing to the stability or acceleration CRC progression is aligned on the left and right colon respectively. Pre-existing tumor immunity, termed “immune contexture”, monitored by “immunoscoring” as well as transcriptome deconvolution represent strong and independent predictors of long-term progression free and overall survival in CRC. Tumors enriched with cytotoxic CD8^+^, CD4^+^, in particular Th1 and Tfh, and B cells, are associated with an IFN-γ response, the upregulation of immuno-inhibitory molecules and better clinical outcome (left). Other types of inflammation, characterized by IL-17 expressing T cells, FOXP3^hi^ Tregs and immunosuppressive myeloid populations are associated with worse clinical outcome. The composition of the ileal microbiome contributes to shift the balance between Tfh and Th17 cells.

CRC outcomes are not only dictated by genetic features but also by the immune contexture (**Figure 2**). Several cell types associated with innate and adaptive immune responses cooperate and dictate the prognosis of patients diagnosed with CRC. γδT cells expressing a heterodimeric T-cell receptor (TCR) are often enriched in epithelial barriers of various mucosae to sense cellular stress at portal of entry (16). However, preclinical murine models of colitis and clinical CRC data have shown that the γδT17 cell subset, producing the IL-17A or IL-17F cytokines, promotes tumor progression through the accumulation of myeloid-derived suppressive cells (MDSC) (17, 18). MDSCs accumulate in the tumor microenvironment (TME), as compared to the adjacent healthy tissue, in patients with CRC and their circulation correlates with cancer stage and metastasis (19, 20). Moreover, Th17 cells through the secretion of IL-17A and the transduction of the STAT3 pathway lead to the downregulation of CXCR3 expression on CD8^+^ T cells. Consequently, these Th17 cells dampen the CXCL10-dependent recruitment of cytotoxic CD8^+^ T cells (CTLs) in advanced stages of CRC (21). In addition, the IL-17R signaling in tumor cells blunts CXCL10 release thereby limiting CTLs influx in tumor bed (22). Furthermore, Th17 cells secrete IL-22 which promotes colitis associated with CRC (23). Contrasting with γδT17 and Th17 cells, IFN-γ producing conventional CD4^+^ T cells, namely, Th1 lymphocytes, are associated with a favorable prognosis in CRC (24).

Another subset of auxiliary T cells, the T follicular helper (Tfh) CD4^+^ lymphocytes, defined by CXCR5 chemokine receptor expression and the Bcl6 transcription factor, are found within and around CRC tumor nests and tumor draining lymph nodes (tdLN). Their density is negatively correlated with CRC tumor progression (25, 26). A positive Tfh/B cell signature associated with increased CD8^+^ T cell infiltrates has been reported in CRC cases with favorable outcomes (25, 27). The Tfh/B cell dialogue is pivotal to orchestrate CD8^+^ T cell effector functions, which are believed to keep in check CRC at early stages of development (28, 29) and may pave the way to the efficacy of immune checkpoint inhibitors (30). Indeed, B cells, FcγR and Ig as well as Th2 and IL-4 have been associated with chronic inflammatory processes, leading to tumor development in T cell-dependent (31–33) or -independent tumor models (34). Likewise, bladder tumors, amenable to therapeutic immune checkpoint blockade, have a dismal prognosis when their TILs contain CD8^+^ T cells producing IL-10 and expressing inhibitory receptors (TIGIT, Lag3, Tim3, and CTLA-4), associated with GATA3^+^ CD4^+^ Th2 cells and Treg (35, 36). Moreover, PD-1 expressing B cells are memory B cells exerting suppressive activity that can be alleviated by anti-PD-1 Abs (37). Another report described PD-1^+^ B cells that possessed regulatory capacity toward T cell responses, and although rare in peripheral blood, they were found enriched in thyroid tumors (38).

However, recent accumulating evidence shows that B cells can orchestrate favorable TCF7^+^ naive/memory CD8^+^ T cell-based immune contexture, specifically when tertiary lymphoid organs can be formed to prime naive CD8^+^ T cells in human tumors, paving the way to clinical benefit to anti-PD-1 Abs (39–41). In breast tumor models genetically modified to express neoantigens, CD8^+^ T cell effector memory cells could be elicited with anti-PD-1 Abs when Treg were depleted (using a mouse anti-CTLA-4 Ab with ADCC properties), therefore triggering a cascade whereby B cells became activated, capable of triggering Tfh activation and IL-21 production. B cell depletion or inhibition prevented class switching, plasma cell generation, and the release of anti-tumor IgG indispensable for tumor rejection following therapy with ICBs (30). In these models of combined immune checkpoint inhibitors (ICIs), effector Tc1 CD8^+^ T cells cannot be generated in the absence of Tfh, IL-2, or B cells. Hence, the optimal B cell activity and therapeutic benefits derived from anti-PD-1/anti-CTLA-4 therapy correlated with concurrent Treg inhibition and Tfh activation (30) or, as described in our study, by lamina propria IL-12 and IL-1β producing DC after stimulation with immunogenic ileal commensals (26).

Tumor-associated Tfh secrete CXCL13, attracting CXCR3^+^ CTLs (42) and *via* IL-21 secretion promotes their effector functions (43). Tfh are involved in the orchestration of the humoral responses through interactions with B cells. Nevertheless, the Tfh/B cells/CTLs collaboration is limited by an increased expression of PD-1 on Tfh during tumor development, dominated by a PD-L1-associated immunosuppression (43, 44). It has been shown that germinal center B cells and/or plasma cells play a protective role, while the T cell suppressive function of regulatory B cell subsets was associated with the dissemination of metastases (45–49). The CD8^+^ Tfh counterpart has also been described in the tdLN of CRC. CXCR5^+^ CD8^+^ T follicular cytotoxic cells (Tfc) express high levels of effector molecules and convey a better prognosis in CRC (50). In line with these contentions, Roberti et al. have recently described the beneficial role of antibody producing B cells in cancer immunosurveillance in murine transplantable colon tumors (26). Hence, as described for other tumor types, terminally differentiated memory B cells and/or plasma cells and Tfh could predict increased patients survival in CRC (49, 51, 52).

*Pagès and Galon* (**Figure 2**) were the first to report the major role of cytotoxic and memory T lymphocytes within primary tumors in predicting survival of CRC patients (28, 53, 54). These studies included early-stage cancer patients (55) and described distinct cellular and molecular cues modulating tumor-infiltrating lymphocyte (TIL) densities (56). A scoring system ‘‘Immunoscore’’ based on the quantification of CD3 and CD8 in the core (CT) and at the invasive margin (IM) of primary tumors has a prognostic value superior to the AJCC/UICC TNM-classification (28, 54, 57, 58). Immunoscore predicts survival of non-metastatic patients, as does the immune infiltrate evaluation in the metastasis for metastatic patients (59–61). These authors have also demonstrated the superiority of Immunoscore over microsatellite instability and PD-L1 expression in predicting survival (62, 63). Focusing on metastasis profiling in CRC, *Galon’s* group brought forward evidence that the cytotoxic T lymphocytes-based adaptive immune response plays a key role in preventing tumor recurrence and metastatic dissemination; despite evidence for clonal expansion of distinct T lymphocyte subsets culminating in immunoediting (60, 64). Disease-free survival (DFS) and OS in stage IV patients are largely governed by the state of the local adaptive immune response within the metastases being the most likely site of tumor immune escape (63). However, further studies are ongoing to better understand how dynamics of genomic and immune patterns shape the CRC metastatic landscape.

### Recent Reclassifications of Colon Cancers

Given the potential significance of the intertwined relationships between genetics and immunometrics, the Cancer Genome Atlas Consortium (TCGA) proposed, in 2012, a different stratification of CRC (65). Four consensus molecular subtypes (CMS1-4) have been discussed to classify CRC based on transcriptomic of the tumor microenvironment (TME) (**Figure 1**) (66, 67).

The CMS1 group (14% of early-stage CRC) showing hypermutation, hypermethylation, and high antigenicity (neoepitopes/neoantigens) is heavily immune infiltrated and predominately constituted by MSI-H tumors (76%). *BRAF* mutations are often found in the CMS1 subtype. The CMS1 group is characterized by a higher expression of immune checkpoints conveying a dismal prognosis (67–70) yet potentially favoring the relative efficacy of immune checkpoint inhibitors (ICIs) (69). CMS2 “canonical” and CMS3 “metabolic” groups (37% and 13% of early-stage tumors, respectively) correspond to “immune-neglected” CRC, in that CMS2 represents an “immune desert” and CMS3, an “immune excluded” tumor microenvironment. The CMS2 group shows *WNT* and *MYC* activation. The CMS2 “immune-desert” subtype is characterized by a poor intratumoral T cell infiltrate. After relapse, the CMS2 group has a superior survival rate than the CMS1 (67). Compared to CMS2, the CMS3 “immune-excluded” subtype often harbors *KRAS*-activating mutations associated with memory Th and naïve B cell-based immune components (71). The CMS4 “mesenchymal” subtype (23% of early-stage tumors) exhibits the prototypic fingerprint of the epithelial-mesenchymal transition (EMT) characterized by a stroma-related gene transcription centered by the transforming growth factor-β (TGF-β) metagene. Stromal cells secret immunosuppressive chemokines that interact with cancer cells and inhibit cytotoxic immune cells, thus contributing to the proliferation of MDSCs (66). CMS4 has the worst relapse-free and overall survival (67). A small fraction of CRC tumors have indeterminate features (13%), and possibly represent a transitional state, or result of a mixed phenotype, due to intratumoral heterogeneity (67).

Recently, based on TCGA transcriptomic analysis of more than 10.000 solid primary tumors, Thorsson et al. have identified six immune subtypes (I.S.) of the tumor microenvironment (C1-C6), namely: wound healing (C1), IFN-γ dominant (C2), inflammatory (C3), lymphocyte depleted (C4), immunologically quiet (C5), and TGF-β dominant (C6). Across all cancer types, the C3 “inflammatory” I.S. was associated with the best overall survival. C1 and C2 I.S. were characterized by a high proliferation rate and exhibited less favorable prognosis even if a higher lymphocyte signature ameliorated the clinical outcome (72). The “wound healing” (C1) and “IFN-γ dominant” (C2) subtypes are the two main I.S. represented in CRC (72, 73). The C1 “wound healing” I.S. showed a prominent expression of angiogenesis-related genes and a low Th1/Th2 ratio. The C2 “IFN-γ dominant” I.S. has the highest intratumoral heterogeneity; containing higher levels of TILs and Tfh cells (73). Nevertheless, the C1 “wound healing” I.S. harbors a better prognosis than the C2 “IFN-γ dominant” I.S. (5-year overall survival (OS) 65% and 49%, respectively), perhaps in line with the upregulation of PD-L1, PD-1, CTLA-4, IDO1, and LAG3 molecules related to immune exhaustion in C2.

### Clinical Management of Colon Cancers

Depending on the TNM score, the main treatment for colon cancers remains the surgical resection of the tumor; especially for tumors with a low risk of recurrence (74). For tumors with stage III or high-risk stage II, a (neo) adjuvant therapy based on chemotherapy is performed prior to surgery to reduce tumor burden and recurrence rates (74). For stage III or high-risk stage II CRC, adjuvant chemotherapies with FOLFOX (5-FU/leucovorin/oxaliplatin) is the standard of care (75–77). As for metastatic diseases, a form of immunotherapy called immune checkpoint inhibitors (ICIs) has transformed the standard-of-care of cancer patients (78–81).

Six months of adjuvant chemotherapy frequently benefit immunoscore-high-patients with stage III primary CRC (82). Moreover, patients treated with chemotherapy and anti-EGFR (Epidermal Growth Factor Receptor) had greater infiltration of T lymphocytes at the core of the metastatic lesion and a higher Immunoscore than patients who received chemotherapy and anti-VEGF (Vascular Endothelial Growth Factor) (63). Hence, Immunoscore^®^ is improved by chemotherapy and predicts the beneficial effects. Oxaliplatin (OXA), a platinum salt used as a cornerstone chemotherapy for CRC (77) is one of the best cytotoxicants capable of inducing immunogenic cell death (ICD) (83). ICD triggers an endoplasmic reticulum (ER) stress response and the activation of the autophagic machinery culminating in tumor cell surface exposure and/or secretion of the mandatory damage-associated molecular patterns (DAMPs); stimulating anticancer immune responses (84). Moreover, OXA increased Tfh density in tumor nests and eventually TILs accumulation as a function of its capacity to induce ileal crypt apoptosis (26).

In CRC, MSI status constitutes a predictive biomarker for response to ICIs. Metastatic CRC with MSI-H phenotype show a high disease control rate and a favorable progression-free survival (PFS) after anti-PD-1 antibody while the 85% patients presenting with microsatellite stable (MSS) or proficient MMR (pMMR) CRC have failed to benefit from various immunotherapy approaches (85, 86). POLE proofreading domain mutations are also associated with a favorable response to ICIs (87). Hence, anti-PD-1 therapies, such as nivolumab and pembrolizumab, have been approved by the U.S. Food and Drug Administration (FDA) in 2017 for patients with MSI-H/dMMR metastatic CRC that have progressed following treatment with a fluoropyrimidine, oxaliplatin, or irinotecan-based chemotherapy (88, 89). Though, the response rate after anti-PD-1 antibodies often remains less than 50% (85, 90), in relation to tumor heterogeneity within MSI-H CRC (91), or immune evasion based on alterations in the antigen processing and presentation machinery (92). However, the immunoscore turned out to exhibit a superior prognostic value for overall survival regardless of the MSI/MMR status. MSI-H patients with a low immunoscore do not have any survival benefit compared with MSS patients. Additionally, in MSS CRC cases, the immunoscore correlates with higher disease-free survival and overall survival (54). Actually, there is no prospective study confirming the clinical significance of the immunoscore for ICI response yet. An ancillary study coinciding with a clinical trial in metastatic MSS CRC (the POCHI trial: NCT04262687) just started to analyze the predictive value of the immunoscore for the clinical benefit to expect from chemotherapy and immunotherapy (93).

The degree of microsatellite instability (MSI) and resultant tumor mutational burden (TMB) have also been shown to underlie the variable response to PD-1 blockade in dMMR, with indel mutations strongly associated with objective response (85, 94). In one of the largest number of patients treated with ICI analyzed for TILs and TMB, Loupakis et al. found that TILs correlate to TMB and the higher the number of TILs, the better the outcome (95). Thus, pembrolizumab is FDA approved for TMB-high tumors (96). In other cases, such as in dMMR/MSI-H metastatic CRC, the combination of ICIs (such as PD-1 and CTLA-4 co-blockade) are likely to be more effective than monotherapy (97). The vast majority of CRC cases, MSS or pMMR CRC, are naturally resistant to immunotherapy; at least in part due to the low antigenicity (TMB) of the malignancy. However, based on TCGA dataset analysis, a small subset of patients among MSI-Low/MSS-CRC cases exhibiting a high CD8^+^ T cell infiltrate and an upregulation of IFN-γ could benefit from immunotherapies (98).

To overcome the lack of efficacy of ICIs in MSS and pMMR CRC, investigators have combined ICIs with chemotherapy and targeted therapeutics (99). Despite these efforts, the combination of oxaliplatin and pembrolizumab has failed to prove superior over OXA-based chemotherapy alone in advanced CRC (100).

Taken together, CRC is the first neoplasia found to be under immunological control, and the first to be treated with OXA, a chemotherapy endowed with immunogenic cell death properties. However, we can see that most attempts to overcome this malignancy have failed, with the exception of a minority of lesions characterized by MSI. This paradox may be solved by a more complete understanding of the role played by the macroenvironment in which this cancer develops (**Figure 1**).

## The Gut Microbiome and the Intestinal Immune System

The gastrointestinal (GI) tract is the major mucosal surface of the human body and the most densely colonized organ. The overall bacterial load of the GI tract has been described to contain between 10^13^-10^14^; nearly equaling the number of the mammalian cells in the body (101, 102).

In mammals, and more specifically in humans, colonization by the intestinal microbiome occurs rapidly during and after birth (103, 104). Successful colonization is determined by microbial selection and competition that begins in the first hours of life (105). Throughout our lifetimes, the microbial population in the human GI tract is affected by a multitude of environmental factors such as age (103, 106), geography (106), dietary habits (107, 108), use of antibiotics (109, 110), host genetics (111, 112), and the pressure of the immune system (113).

The intestinal microbial ecosystem has a significant role in host physiology in multiple ways. The processing of food and xenobiotics enables a host to obtain essential nutrients (101). The intestinal microbiota promotes post-natal maturation of physiological gut functions such as the integrity of the epithelial barrier, the expression of essential enzymes (114), the development of the intestinal vascularization (114, 115), and of the enteric nervous system; all essential for motility (116). Most importantly, the gut microbiota participates in the maturation of the local and systemic immune system to ensure tolerance *vis-à-vis* of food antigens and the elimination of pathogens, maintaining a mutual symbiosis between commensals and self-tissues for the homeostasis of the meta-organism (117, 118).

**Figure 3:**
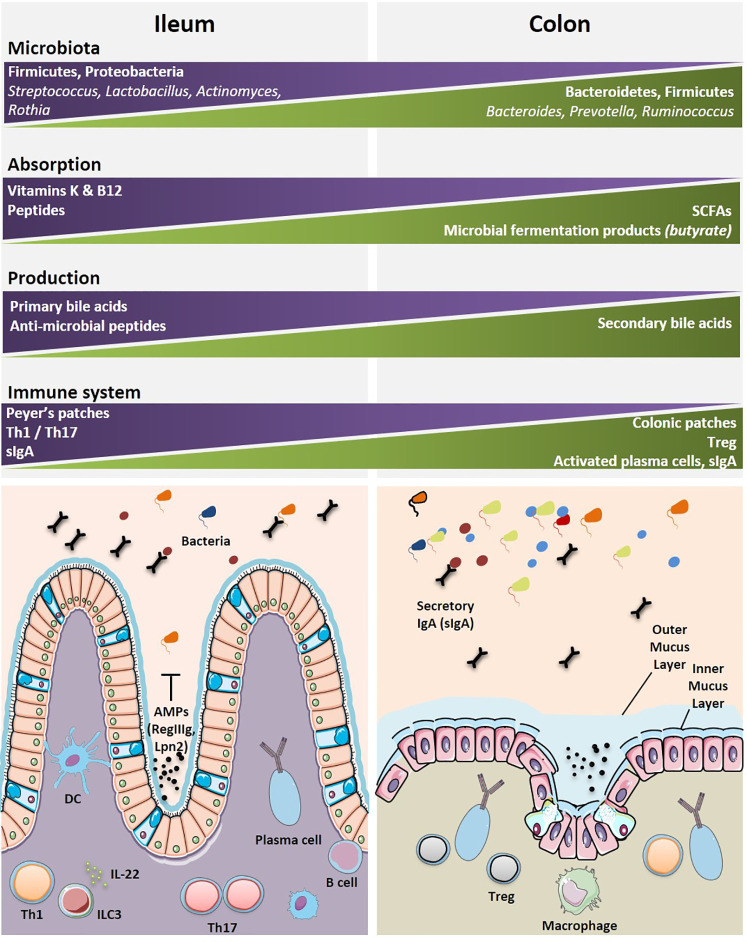
Geographical distributions of immune and microbial components highlight specificities between the ileal and colonic segments. The most dominant microbial and immune cells accounting for the diversity and specificity of each organ are depicted. The main specialized functions (metabolic, immune, motility, etc.) for each segment are indicated. A graphical abstract is lining below the boxes where these cells and functional specificities are listed. Immune cells are found either clustered within organized lymphoid structures or scattered within intestinal epithelium (IE) and lamina propria (LP). AMP, anti-microbial peptides; B cell, B cell lymphocyte; DC, dendritic cell; ILC3, innate lymphoid cell type 3; IL-22, interleukin-22; Th1, T helper 1 cell; Th17, T helper 17 cell; Treg, regulatory T cell.

Various bacterial communities are disseminated along the GI tract with diversity affected by anatomical and environmental differences from the esophagus to the rectum. There is a limited diversity of the microbiome in the esophagus where *Streptococci* remain the dominant species (119). Similarly, the stomach has limited microbial diversity due to low pH of the gastric lumen (120). While the small intestine and colon harbor a wider commensalism compared to that of the upper GI tract. Different physiologies and environments such as chemical, oxygen and nutrient gradients, and the compartmentalized immune system along the GI tract result in distinct distribution of the intestinal microbiota (121). The small intestine contains a smaller load of bacteria and with less diversity, than the colon. This is due to a number of factors such as harsh environment for bacterial communities, a shorter transit time, an increased influx of digestive enzymes of antimicrobial peptides and bile acids, and an intermittent food substrate delivery (121, 122). Although the taxonomic classification has been inconsistent across studies, several reports show the predominance of two major phyla, i.e., Firmicutes and Proteobacteria (121, 123, 124), followed by others (Bacteroidetes, Fusobacteria, Verrucomicrobia, Actinobacteria) residing in the human small intestine (124). At the genus level, several genera are commonly found in the small intestine, such as *Lactobacillus*, *Clostridium*, *Staphylococcus*, *Streptococcus*, and *Bacteroides* (124–127). Sample collection from the distal ileum has shown that *Streptococcus, Granulicatella, Actinomyces, Solobacterium, Rothia, Gemella*, and TM7(G-1) are the most frequently detected bacterial genera by 16S gene sequencing (127) with *Streptococci*, *Actinomyces, Rothia* and *Lactobacillus* species most frequently identified by culturomics and mass spectrometry (128). In contrast, the colon contains roughly 70% of all the bacteria of the human body (129). The microbial population in the colon is a result of environmental factors such as lower concentrations of antimicrobials, slower transit time, and fermentation of polysaccharides (121, 129). Interestingly, the two major phyla in the colon are Bacteroidetes and Firmicutes (121, 129). At the genus level, *Bacteroides*, *Prevotella*, and *Ruminococcus* are predominant (129) (**Figure 3**).

Fecal profiling of the microbial populations is investigated most frequently because the sampling of feces is non-invasive and convenient for patients compared to performing an invasive biopsy or collecting luminal content of ilea or colons (130). However, there is increasing evidence that the observed microbial composition in feces is different from the content of mucosal samples of the GI tract (123, 131–135). Bacteroidetes and Firmicutes are the main phyla in the healthy human stool samples followed by other phyla such as *Actinobacteria*, *Proteobacteria, Synergistetes*, and *Verrucomicrobia* (123, 134, 136–142). At the genus level, *Bacteroides* is the most abundant genus (136, 140, 141) followed by *Faecalibacterium*, *Bifidobacterium*, *Lachnospira*, *Roseburia*, *Subdoligranulum*, *Collinsella*, *Ruminococcus*, *Prevotella*, *Alistipes*, and *Akkermansia* (123, 132, 136–142). Of note, mucosal microbiome of the colon differs from that of fecal composition. James et al. reported that *Enterococcus* was found to be more prevalent in the proximal colon while *Coprobacillus* and *Escherichia/Shigella* were more abundant in the distal colonic mucosae (143).

The symbiotic relationship between the gut microbiome and the associated local immune system is central for the maintenance of the systemic immune tonus. The lamina propria and gut-associated lymphoid tissues (GALT) contain the largest pool of cells mediating innate and cognate immune responses (**Figure 3**). There is marked regional variation in immune cells along the GI tract, with Th17 decreasing in number from the duodenum to the colon, and regulatory CD4+ T cell (Treg) being most abundant in the colon (144). Pioneering mouse studies demonstrated that distinct bacterial species can fine-tune intestinal immune responses, such as Th17 (145, 146), Treg (118, 147), or Th1 (148, 149), Tfh (148), and B cell activation (150). James et al. catalogued the mucosal microbiome in different regions of the human colon and reported the annotated colon immune single-cell dataset at the Gut Cell Atlas (https://www.gutcellatlas.org/) (143). There are 25 cell types in the lamina propria (LP) and mesenteric lymph nodes (mLNs), draining the healthy colons. Among these, are follicular and memory B cells, IgA^+^, and IgG^+^ plasma cells, effector and memory CD4^+^ T cells, Treg cells, CD8^+^ T cells, γδ T cells, innate lymphoid cells, natural killer cells (NK), mast cells, and myeloid cells (cDC1, cDC2, pDC, LYVE1^+^, or CD16^+^ macrophages, monocytes). B cells, dendritic cells and γδ T cells are Ki67^+^ cycling populations compared to other colonic immune cell populations. The cecum and the sigmoid were reciprocally enriched in CCL20^+^ Th17 and Th1 respectively. While mLN contained follicular B cells and memory CD4^+^ T cells, colonic mucosae, and most specifically the sigmoid LP is rich in effector CD4^+^ T cells and plasma cells. Region-specific transcriptional differences linked activation and tissue migration of Th1 and Th17 cells of the proximal and sigmoid colon as well as the identification of clonal sharing between these colonic regions plead for cell-extrinsic rather than cell-intrinsic factors regulating T cell functions. However, the relative proportion of Treg cells do not change significantly from proximal to distal colon. There is heterogeneity in Treg cell states in the mLNs and colon with a transient loss of the FoxP3-expressing cell population. Data infer a continuous activation trajectory of these Treg cell states between draining lymph nodes and colon, with genes regulating Treg cell migration and adoption of Th-like profiles in tissues (143). Treg transiently losing FoxP3 expression could re-express it transcription under activation to achieve their bona fide immunosuppressive functions (151). There is a highly activated state of plasma cells found in the distal colon compared with proximal colon plasma cells, which are characterized by greater accumulation, somatic hypermutation, clonal expansion, and stronger homing to the colonic mucosa (143). Compared with the cecum, IgA^+^ plasma cells of the sigmoid colon respond to a rich and unevenly represented community of bacterial species; most likely accounting for the increased activation, migratory and greater clonality status of plasma cells (143). Indeed, by means of a novel method for identifying and isolating lymphoid follicles along the length of the human intestine and IgA sequencing analysis, *Agace’s* group showed that IgA adaptive immune responses are initiated in an anatomically restricted manner in the human Peyer’s patches and submucosal isolated lymphoid follicules contributing to the ileal and colonic plasma cell repertoires, respectively (152).

## Dysbiosis Associated or Causally Linked With Colon Carcinogenesis

**Figure 4:**
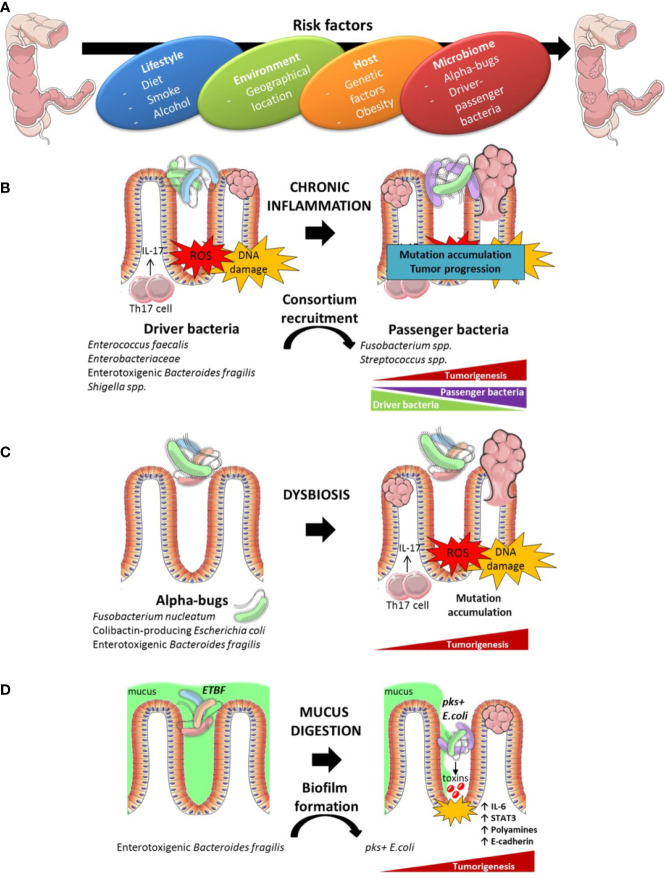
Instrumental links between intestinal dysbiosis and colon carcinogenesis. Risk factors contributing to coloractal cancer (CRC) initiation and development encompass the life style, diet, the exposure to environment (xenobiotics), host genetics and the gut microbiome **(A)**. Several hypotheses have been formulated to account for the toxicity of the microbiome for the epithelium. A driver bacterium could recruit a consortium of disease-facilitating microbes **(B)**. The “keystone hypothesis” suggests that specific bacteria lead to dysbiosis and pro-carcinogenic microenvironment **(C)**. Mucus digestion by some bacteria could expose the epithelium to toxins produced by virulent bacteria organized in biofilms **(D)**.

Western style diets (high fat, high sugar) and meat consumption may in fact be major risk factors in the development of CRC (153). Diet displays a dominating role in shaping the structure of gut microbiota, occasionally leading to a detrimental alteration of microbiota ecology and functionality. Indeed, increasing dietary fiber can lead to the establishment of a favorable microbiota regulating the production of the anti-inflammatory short chain fatty acids (SCFA) (154). An unbalanced microbiota, known as dysbiosis, has been associated with many maladaptive states, including CRC (155–159). (**Figure 4A**) However, the contribution of the microbiota in CRC initiation and development remains a subject of debate. Pre-clinical and human clinical studies have linked the intestinal microbiota to CRC. Pre-clinical studies performed with sporadic CRC rodent models showed that germ-free mice and rats display less intestinal tumorigenesis than animals conventionally reared, highlighting the role of the microbiota in CRC emergence (160). Confirming this functionality, the first mouse model of spontaneous invasive CRC has been described, in which resident microbiota play a key role in disease outcome (161). Indeed, intestinal epithelial cell (IEC)-specific transgenic expression of the epithelial-mesenchymal transition regulator Zeb2 in mice (*Zeb2* ^IEC-Tg/+^ mice) lead to increased intestinal permeability and spontaneous invasive colon carcinoma development in a microbiota-dependent manner (161). However, this work did not yet identify CRC associated- microbial entities. Several independent studies comparing tumor versus paired adjacent healthy tissues showed marked differences in the gut microbiota composition (162–165), with either depletion or enrichment of selected bacterial species. Meta-analysis of five publicly available datasets and two new cohorts with validation using two additional cohorts, considering n=969 and n=768 fecal metagenomes revealed an enrichment of bacterial species within CRC and feces of patients, not found in homeostatic controls. They identified 29 species, mostly from the oral cavity, associated with CRC development (166, 167). At the functional level, the choline trimethylamine-lyase gene (166) as well as protein and mucin catabolism genes were overabundant while carbohydrate degradation genes were depleted (167) in CRC.

Of note, this particular microbial shift distinguishes benign from malignant colon tumors (166). Alterations of microbiota composition are distinct across major stages of colorectal carcinogenesis (168, 169), suggesting a role of specific bacterial communities in this process. Moreover, besides the important shifts observed in the colonic microbiome of CRC, significant variations have been reported in the ileal microbiome. Using healthy ileal mucosa-associated microbiome, lining upstream from Bauhin’s valve and collected during hemicolectomy in patients suffering from different stages of proximal CC, Roberti et al. (26) identified distinct species and family members conveying immunogenicity to CRC by regulating the accumulation of Tfh instead of Th17 cells in the tumor microenvironment.

Furthermore microbiome diversity and richness changes during carcinogenesis, bacteria can be found with a specific organization in normal colon mucosa, mirroring the bacterial composition within the tumor. Bacterial organization in biofilms protects commensals against external agents and induces pro-oncogenic properties by inducing major deregulation of epithelial cell biology culminating in sustained inflammation as detailed below (**Figure 4**). Biofilms are associated with increased risk of developing sporadic CRC (170, 171). Formation of biofilms is mostly a characteristic of right-sided colon cancer (170).

Altogether, these studies showing significant variations of taxonomic footprints suggest a role of the gut microbiota in the emergence of CRC. However, whether one or more species are necessary for CRC development remains unclear. Notable steps forward have been made in the identification of single bacterial species or bacterial communities associated with CRC. Several studies have nailed down mechanistic cues that link distinct bacteria strains and species to CRC carcinogenesis. Bacteria can exert their pro-oncogenic effects through multiple mechanisms. The inflammatory contexture can modulate microbial gene functions and increase the cancer-promoting activity of some bacteria strains, as exemplified for *E.coli* NC101 (172). Bacteria can produce toxins and metabolites from the fermentation of by-products or induce the formation of superoxide radicals that lead to subsequent genetic mutations in the colonic epithelium (173, 174). Several theories have also been proposed to delineate the involvement of specific microbiota in CRC initiation or progression (**Figure 4**). The “driver-passenger” model supports the idea that a microbial leader recruits a consortium of disease-facilitating microbes to initiate the biological events causing CRC (175–177). This model suggests a chronological recruitment of specific bacteria concurring to CRC. First, “driver” bacteria create a pro-oncogenic environment through DNA-damage and malignant transformation of epithelial stem cells. After initiation of tumorigenesis, there is an emergence of “passenger” bacteria, more adapted to the tumor environment such as *Fusobacterium nucleatum* and *Streptococcus bovis/gallolyticus* (177) (**Figure 4B**). Secondly, the ”keystone hypothesis,” or “alpha-bug hypothesis,” suggests that certain low-abundance bacteria possessing unique virulence traits can reshuffle a benign environment into a carcinogenic one. For instance, enrichment of *Fusobacterium* species is associated with a depletion of the Bacteroidetes and Firmicutes, in malignant colon relative to normal colon tissue (175, 178–180). Thus far, several “alpha-bugs” that promote intestinal carcinogenesis in animal models have been described such as *Fusobacterium nucleatum* (181, 182), colibactin-producing *Escherichia coli* (183–185) and enterotoxigenic *Bacteroides fragilis* (ETBF) (186) (**Figure 4C**).

*Fusobacterium nucleatum* is frequently associated with advanced proximal CC tumors, metastasis, chemoresistance, and poor prognosis (26, 187–189). These pathogenic properties occur through the expression of two different virulence factors, FadA, and Fap2 on the intestinal CRC and non-CRC epithelium, which induce the expression of a large spectrum of transcription factors, oncogenes, and genes coding for inflammatory functions or tumor progression (182, 190). *F. nucleatum* shapes the immune environment to promote tumor growth through the elicitation of a pro-inflammatory cytokine cascade (IL-8, CXCL1) and the direct inhibition of T and NK cell functions (191, 192). Furthermore, *F. nucleatum* has been shown to modulate autophagy in intestinal epithelial cells (IECs) by activating regulatory microRNAs that consequently alter colorectal cancer chemotherapeutic responses (189) (**Figure 4**).

Regulatory T cells (Treg) can be anti-tumorigenic or pro-tumorigenic in colorectal cancer (CRC) depending on the presence of different Treg subsets with various immunosuppressive molecules. FoxP3 expression intensity dictates the prognosis of CRC; FoxP3(hi) Treg being associated with poor clinical outcome in contrast to producing-FoxP3(lo) Treg producing inflammatory cytokines (193). Earlier work described non-effector T cells comprising regulatory and anergic T lymphocytes that specifically accumulate in tumor tissues and eventually recirculate (194). Some reports suggested synergistic associations between PD-1/CTLA-4 and PD-1/CD39 within Helios^+^ or Helios^neg^ FoxP3^+^ CD4^+^ T cells to dampen T-cell activation and functions in CRC (195, 196).

*Escherichia coli*, although commonly found in homeostatic gut microbiota, is another bacterium frequently correlated with tumor staging and prognosis (184). The association between *E. coli* and CRC specifically implies *E. coli* strains that produce the genotoxin colibactin. This toxin accelerates tumor progression (185, 197) by damaging host DNA (172, 183, 198) and by inducing cellular senescence (**Figure 4**). This process may involve the production of several growth factors in human CRC (197).

*ETBF* is an enterotoxin-producing bacterium that is thought to play a role in the occurrence and progression of CRC (186). The toxicity of this bacterial compound is linked to its capacity to damage DNA strands *via* the generation of reactive oxygen species (ROS) and through the induction of Th17-dependent inflammatory responses (186). The ability of *Bacteroides fragilis* to promote tumor growth presumably relies on the enterotoxin production, as nontoxigenic *B. fragilis* (NTBF) does not induce Th17 responses and fails to facilitate cancer outgrowth (186), offering prophylactic effects against colitogenic *B. fragilis* (199). While IL-17 initiates a NFκB-mediated recruitment of protumoral CXCR2^+^ myeloid cells (200), other mechanistic events mediated by toxigenic *B. fragilis* contribute to colon tumor development. In particular, digestion of the mucus layer by ETBF enabled enterotoxigenic species, namely pks+ *E. coli*, to adhere to colonic IEC, thus facilitating access to the toxin (201).

Finally, mucus-invasive bacterial biofilms were identified on the colon mucosa of 50% of CRC patients and approximately 13% of healthy individuals. Remarkably, biofilm-positive communities from healthy colonoscopy induced colon inflammation and tumors similarly to biofilm-positive tumor tissues in three independent mouse models of CRC (202). Bacterial genera shown to be amplified in CRC patients such as *Clostridium XI, Clostridium XVIII, Erysipelotrichaceae incertae sedis, Escherichia/Shigella, Eubacterium, and Parabacteroides* were increased in biofilm-positive–associated mice. These bacteria may interact with one another. Collaboration between multiple types of bacteria is likely to be a contributing factor, as suggested by the identification of ETBF and pks+ *E. coli* within familial adenomatous polyposis mucosal biofilms. In contrast, *Bifidobacterium* was depleted in mice inoculated with biofilm-positive tissues, confirming the metagenomics data of stool composition in CRC patients (162, 203). Mechanistically, biofilm-positive colon cancers have been associated with decreased E-cadherin expression, an increased release of IL-6, and the polyamine metabolites N(1),N(12)-diacetylspermine, activation of STAT3, all culminating in IEC proliferation and cell transformation (170) (**Figure 4D**). This work converged into the demonstration that biofilm formation plays a key carcinogenic role (202).

The composition of the ileal microbiome influenced the accumulation of T follicular helper (Tfh) cells and CD8^+^ T cells in proximal colon cancers (26). Cremonesi et al. showed that distinct bacteria species can directly stimulate colon cancer cells producing a chemokine array closely correlating with T cell subsets expressing the appropriate chemokine receptors, which in turn, may modulate tumor immunosurveillance and dictate the prognosis of colon malignancies. Hence, *Fusobacteria* and *Prevotella* can trigger CCL20 release through tumor cells *in vitro*, while Th17 infiltration *in vivo* was associated with “cold” (poor in tumor infiltrating lymphocytes) CRC (204, 205).

Aside from Th1 and Th17 responses, the tumor contexture can also reveal the presence of beneficial Th9 that could be associated with specific local microbes. In recent reports, IL-9 producing-Th9 cells resulted from the conversion of Th2 cells into Th9 cells endowed with pro-apoptotic tumoricidal activity and immunizing capacities (206). Moreover, positive and negative correlations between intratumoral IL-9 and abundance of *Prevotella or Bacteroides* spp. were reported in CRC respectively (207).

Wong et al. also demonstrated a causal relationship between the composition of CRC patients’ stools and carcinogenesis. Wong et al. fed fecal samples from patients diagnosed with CRC (versus healthy volunteers) to germ-free mice and conventional mice treated with the procarcinogenic azoxymethane. CRC-derived feces promoted polyp formation, intestinal dysplasia, epithelial proliferation and stemcellness, as well as inflammation (IL-17A, IL-22, IL-23A) associated with the colonic recruitment of Th1 and Th17 cells (208).

## Colon Cancer Therapies and Gut Microbiota

Most pharmacological compounds in the oncological armamentarium have the capacity to perturb the delicate triangle of fitness and integrity of the intestinal barrier, the microbiota ecosystem, and the gut immune system. Such “off target” side effects of targeted therapeutics drugs may eventually affect their anticancer efficacy and/or the safety profile. Multiple chemotherapies and immune checkpoint inhibitors have been studied in preclinical models of colon carcinoma (26, 209, 210) and pancreatic cancer (211) expanding our current understanding of mechanisms underlying response.

**Figure 5:**
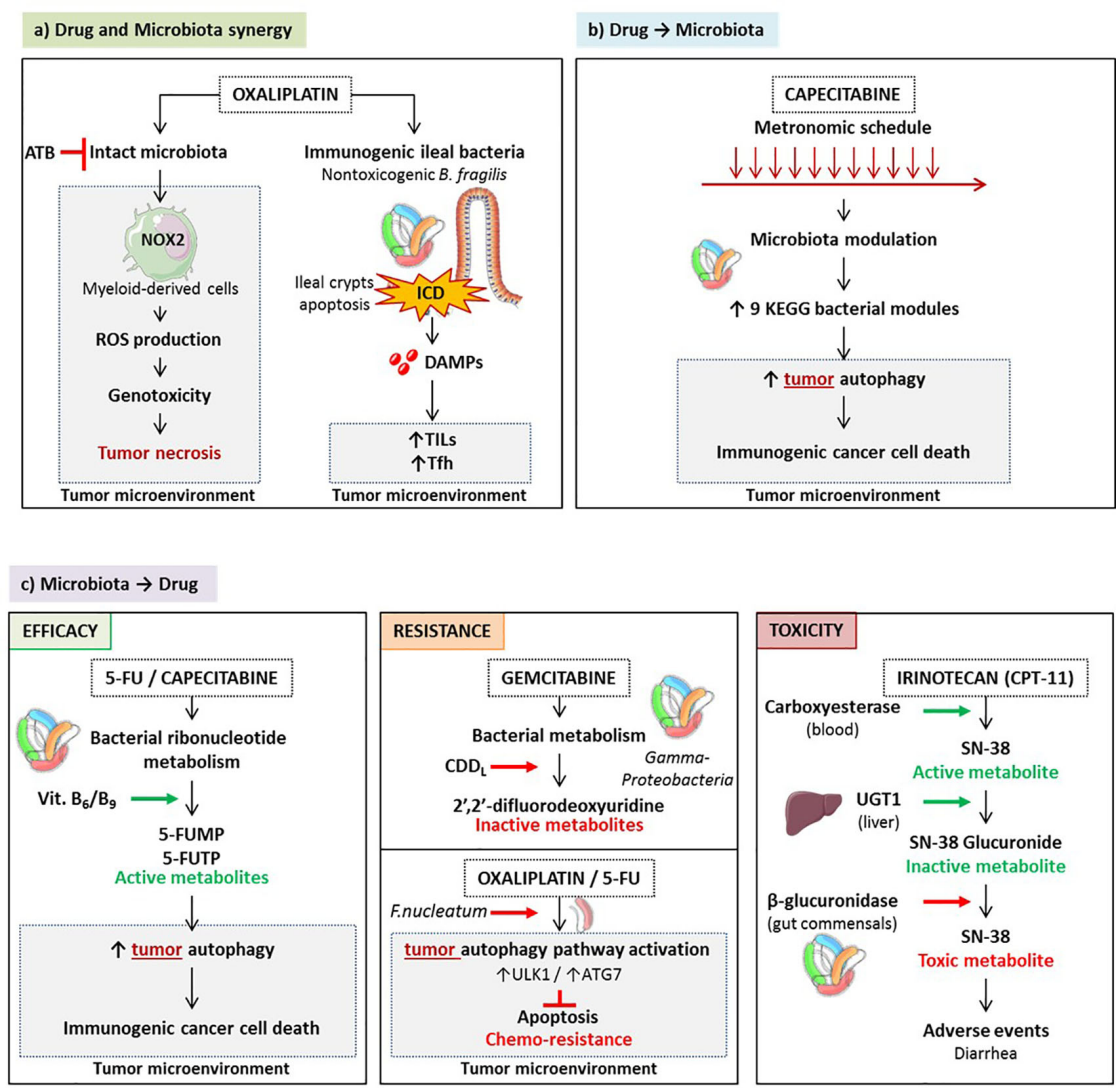
Pharmacological-microbiota interactions. Drugs and microbes can mutually influence each other. A synergy - exemplified by oxaliplatinum - can occur culminating in triggering the local immune system eventually controlling the tumor microenvironment. This synergy is severely compromised by antibiotics. **(A)** Drugs can shape gut microbiota and activate bacterial functions beneficial for the anticancer therapy. For instance, CTLA-4 blockade could enrich the ileal microbiome with distinct *Bacteroides* spp. that in turn fosters Th1 responses beneficial against cancer. **(B)** On the other hand, commensals can metabolize anticancer prodrugs, thus activating or inactivating their bioactive metabolite, sustaining or compromising the therapeutic effect. Conversely, increased recycling of bioactive compounds may be accelerated by enzymatic machineries associated with distinct microbes (such as camptothecin-11), generating severe side effects. **(C)**.

Oxaliplatin (OXA) is a clinically effective tumoricidal platinum salt commonly used against colon, breast, ovarian and lung carcinomas (212, 213). The initial mode of action described for OXA was that it creates DNA damage, inducing formation of intra- and interstrand DNA adducts, generated by crosslinking between activated platinum species and specific base sequences (214). However, in the absence of a functional immune system, in particular a TLR4 signaling pathway and T lymphocytes, OXA fails to mediate full blown anticancer activity in tumor bearing mice and in patients diagnosed with CRC (215, 216). Indeed, following OXA-based chemotherapy, antitumor adaptive immune responses are activated (217). This activation occurs through the induction of immunogenic cell death (ICD) of colon cancer cells due to the initiation of executioner caspases (216, 218–220) and through the subsequent release of several damage associated molecular patterns (DAMPs) including surface exposed-calreticulin (220), high mobility group box 1 protein (HMGB1) (221), CXCL10 (223), and Annexin A1 (224). Moreover, the antitumor efficacy of OXA largely depends on the presence of an intact microbiota. Disruption of murine microbiota with broad-spectrum antibiotics was shown to reduce the cytotoxicity and production of reactive oxygen species (ROS) *via* the NADPH oxidase NOX2 by tumor-infiltrating myeloid cells after OXA treatment (209). ROS are needed for the OXA antitumor effect. Although the genotoxic effect of platinum compounds was known to require ROS and particularly H_2_O_2_ production by tumor cells *in vitro*, ROS is produced by tumor-associated myeloid cells *in vivo*. Thus, the microbiota affects OXA early tumor genotoxicity by systemically priming tumor-associated myeloid cells for ROS production (209) (**Figure 5A**).

Finally, OXA does not just induce apoptosis of tumor cells. Chemotherapy can promote apoptotic cell death in the intestinal crypts of healthy tissues, known as chemotherapy-induced mucositis (224). As many other anticancer agents, OXA compromises the integrity of the intestinal barrier which may create a dysbiosis and potentiate systemic inflammation and immunity (225–228). It has been shown that OXA is associated with cell apoptosis in ileal, but not colonic, crypts sparing the villi and the lamina propria, in tumor bearing mice and in patients diagnosed with proximal colon cancer (pCC) (26). Ileal crypt apoptosis was correlated with progression-free survival and the accumulation of TILs and Tfh in the tumor bed (26). (**Figure 5A**) Ileal cell apoptosis requires caspase 3 and caspase 7 executioners as well as ileal commensals. First, tumor bearing mice in which caspase 3/7 expression was ablated specifically in intestinal epithelial cells (IEC) (*Casp3/7* ^ΔIEC^) failed to respond to OXA while RIPK3-deficient animals disabled for necroptosis of the IEC controlled tumor outgrowth during OXA therapy (26). Secondly, when broad spectrum antibiotics were administered along with OXA, the apoptosis of the crypts was blunted and the anticancer effects were abrogated. To consolidate the impact of patient microbiota on the efficacy of OXA, Roberti et al. utilized an avatar model (229), in which the intestines of germ free mice were colonized with human colonic content collected from pCC patients. Two weeks later, these mice were inoculated with subcutaneous MC38 and 7 days later, treated with OXA. While 66% of patient microbiota resulted in antitumor efficacy of OXA comparable to that observed in mice reared in specific pathogen-free conditions, 33% patients’ microbiota induced complete resistance to this immunogenic chemotherapy. Ileal crypt apoptosis was highly correlated with major changes in the composition of the ileal microbiome, with a dominance of *Erysipelotrichaceae* at the expense of *Fusobacteriaceae.*

Capecitabine, an oral prodrug of fluorouracil, inhibits the enzyme activity of thymidylate synthase during DNA replication (230). It has been proven effective in conjunction with OXA in metastatic colon cancers and other cytotoxicants in breast cancer and is widely used in adjuvant setting. A pioneering study explored the role of microbes in modulating the effect of 5-FU and other fluoropyrimidines on *Caenorhabditis elegans* and found that bacteria are key determinants of fluoropyrimidine efficacy on host metabolism (231). Microbes can boost or suppress the effects of fluoropyrimidines through metabolic drug interconversion involving bacterial vitamin B_6_, B_9_, and ribonucleotide metabolism. (**Figure 5C**) Also, modulations in bacterial deoxynucleotide storage amplify 5-FU-induced autophagy and cell death in host cells. A Chinese prospective study of the fecal composition of 31 females treated for HER2 negative breast cancer used 16S rRNA sequencing to analyze the variations of the gut microbiota during a maintenance chemotherapy comparing metronomic versus conventional dose of capecitabine (232). While alpha diversity was not different between the two treatment modalities, beta diversity varied significantly between the two groups, with a relative depletion of *Cyanobacteria*, *Chloroplast, Blautia*, *and Streptophyta* in stools of the metronomic capecitabine -treated females. Multivariate analyses of progression-free survival of all 31 patients regardless of capecitabine dosing revealed that *Blautia obeum* was significantly associated with clinical benefit (HR 3.4) in contrast with *Slakia* (HR 0.2). For the study of relative bacteria functions, the Kyoto Encyclopedia of Genes and Genomes (KEGG) modules were quantified, indicating that metronomic capecitabine tended to enrich for bacteria involved in nitrification, and putrescine, lysine/arginine/ornithine transport system. These metabolic traits are reminiscent of polyamine synthesis closely linked with the activation of the autophagic machinery mandatory for the immunogenicity of cancer cell death (233) (**Figure 5B**). Although concerning for breast cancer females, and underpowered, this pioneering study suggests that distinct chemotherapeutic regimen influence the composition of the gut microbiota that in turn, could impact clinical outcome (232).

### Gemcitabine and Camptothecine

Bacteria can metabolize chemotherapeutic drugs, to increase or decrease their pharmacological effect. The presence of bacteria inside human tumors may paradoxically result in drug concentrations that are lower in the tumor than in other organs. Gemcitabine is a nucleoside analog (2′,2′-difluorodeoxycytidine) used to treat patients with pancreatic, lung, breast, or bladder cancers. It has been occasionally combined with FOLFOX against colon cancer (234). Bacteria can metabolize the chemotherapeutic drug gemcitabine (2′,2′-difluorodeoxycytidine) into its inactive form, 2′,2′-difluorodeoxyuridine (211). Metabolism is dependent on the expression of a long isoform of the bacterial enzyme cytidine deaminase (CDD_L_), seen primarily in *Gammaproteobacteria* found in pancreatic ductal adenocarcinoma or colon cancers (211). In a colon cancer mouse model, the authors demonstrated that gemcitabine resistance was induced by intratumor *Gammaproteobacteria*, dependent on bacterial CDD_L_ expression, and abrogated by the ciprofloxacin fluoroquinolone. Antibiotic-treated mice displayed a better antitumor response to gemcitabine than control mice (211) (**Figure 5C**).

Bacteria could confer drug resistance through the induction of autophagy in colorectal cancer cells, therefore interfering in the tumoricidal activity of chemotherapy. *F. nucleatum* is gradually increased from normal tissues to adenoma tissues and to adenocarcinoma tissues in colorectal carcinogenesis (178, 179). Moreover, the amount of *F. nucleatum* in CRC tissues was associated with shorter survival (187). Another independent group found that the five-year recurrence survival was substantially shorter in patients with CRC rich in *F. nucleatum* than in those poor in *F. nucleatum* (189). Multivariate regression analyses demonstrated that the amount of intratumor *F. nucleatum* was an independent predictor of CRC aggressiveness and recurrence post-chemotherapy with significant hazard ratios for predicting clinical outcome (189). ULK1/ATG7 -dependent autophagy contributed to *F. nucleatum*-mediated CRC resistance to OXA and 5-FU regimens (189). *F.nucleatum* induced- genomic loss of miR-18a* and miR-4802 depended upon the TLR4/MYD88 signaling pathway (189) (**Figure 5C**).

Camptothecin, a potent antineoplastic compound, poisons the catalytic cycle of human topoisomerase I. Camptothecin exhibited marked toxicity and poor bioavailability. Although its derivatives topotecan and CPT-11 (also called irinotecan) are now in clinical use (234), they still elicit pronounced side effects that limit efficacy. CPT-11 is one of the three commonly used chemotherapeutic agents for CRC (236). It is a prodrug that gives rise to the active metabolite SN-38 *in vivo* (237). Intravenously administered CPT-11 is activated by carboxylesterases to SN-38, the antineoplastic topoisomerase I poison. Liver SN-38 is inactivated *via* glucuronidation to SN-38G by UDP-glucuronosyltransferases and spread to the GI tract. β-Glucuronidases in the gut commensals remove the glucuronide as a carbon source, and active SN-38 in the intestinal lumen generates dose-limiting diarrhea. High throughput screening of inhibitors selectively targeting the enzyme in living bacteria, and sparing non relevant bacteria and mammalian cells led to the identification of promising compounds that could reach a better therapeutic index of camptothecin-11 (238) (**Figure 5C**).

### Immune Checkpoint Inhibitors (ICIs)

**Figure 6:**
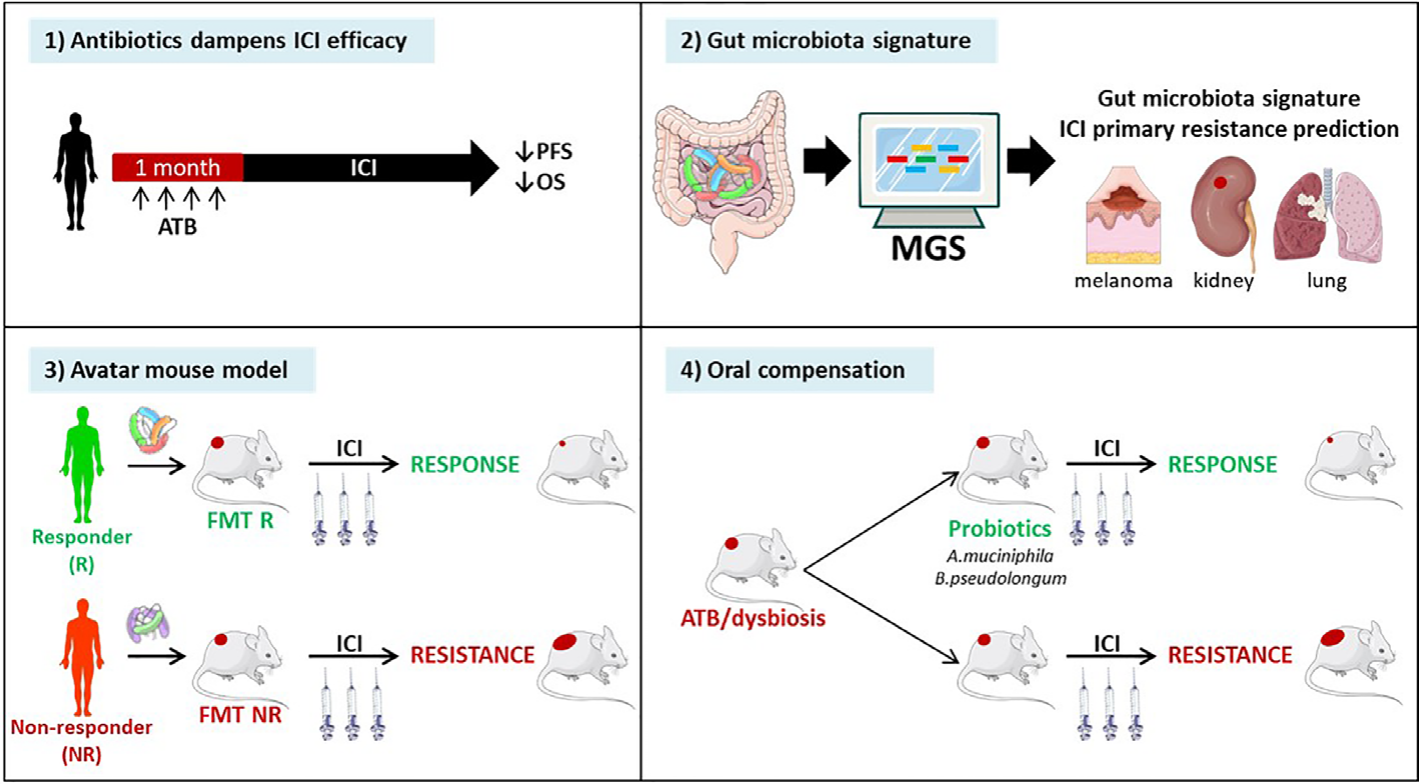
Evidence pointing to a key role of the gut microbiota in immune checkpoint inhibitors (ICI)-mediated anticancer effects. Recently, pre- and clinical studies have shown the clinical significance of the composition of the gut microbiota in ICI efficacy. 1) Retrospective and prospective studies have highlighted the detrimental effect of antibiotics administration within the month preceding the start of immunotherapy in multiple stage III and IV cancers. 2) Metagenomic analysis (MGS) of patients’ stool composition predicts primary resistance to ICI in 1L and 2L melanoma, kidney and lung cancers. 3) Avatar mouse models where the intestines of tumor bearing rodents are colonized by human stools from cancer patients at diagnosis allowed to predict the response to immunotherapy in patients. 4) The identification of beneficial bacteria, such as *Akkermansia muciniphila* or *Bifidobacterium pseudolongum* for immunotherapy efficacy opens up prospects to restore cancer-associated dysbiosis in patients.

DNA mismatch repair-deficient or microsatellite-instable tumors particularly benefit from the immune checkpoint blockade (85). Accumulating evidence points to the critical role of the gut microbiota in the efficacy of ICIs. First, antibiotics taken within the month preceding start of anti-CTLA-4 or anti-PD-1 antibodies, markedly attenuated the clinical benefit, reducing both progression-free and overall survival across many metastatic and stage III malignancies amenable to therapies based on immune checkpoint inhibition (239, 240). Second, fecal microbial transplantation of stools from patients prone to respond to ICI or doomed to fail first or second line ICI-based therapy confer sensitivity or resistance to tumor bearing mice treated with anti-PD-1 antibodies respectively (241). Third, shot gun metagenomics sequencing of patients’ stools at diagnosis may help predicting primary resistance to PD-1 blockade in metastatic melanoma (242), kidney (241) and lung cancers (243). Fourth, oral compensation of antibiotics-treated tumor bearers or dysbiotic hosts with monoclonal anticancer probiotics (such as *Akkermansia muciniphila* (243), or *Bifidobacterium pseudolongum* (244) restored full blown immunostimulatory activity of anti-PD-1 antibodies. (**Figure 6**). Conversely, ICIs also have an impact on the gut composition. Vetizou et al. reported that anti-CTLA-4 antibody modulated the ileal bacteria composition, increasing the relative abundance in *Bacteroides* spp. (namely *B. fragilis*) and Burkolderiaceae family members, involved in the IL-12-dependent priming of Th1 cells as well as the immunostimulatory and anti-cancer effects of CTLA-4 blockade (210). In line with these preclinical data, six months of therapy with nivolumab ameliorated the alpha diversity of metastatic kidney cancer bearing patients in those individuals benefiting from the antibodies (241). Such studies are awaited in MSI^high^ CRC amenable to PD-1 blockade. Finally, memory Th1 and Tc1 immune responses directed against immunogenic bacteria *E. hirae, A. muciniphila*, and *B. fragilis* dictated progression free survival in cancer patients treated with anti-PD-1 or anti-CTLA-4 Abs (210, 243).

## Towards a Solution to the Paradox of Colon Cancers

As stated above, the reasons why DNA mismatch repair-proficient or microsatellite-stable CRC, endowed with tumor infiltrating lymphocytes at the primary or metastatic stage and treated with a cytotoxic compound mediating ICD, fail to benefit from immune checkpoint blockade are enigmatic (85, 100). Indeed, FOLFOX could induce T-bet-dependent PD-1 expressing CD8^+^ T cell infiltration and IFN-γ-mediated PD-L1 upregulation in mouse models of colon cancers, and in CRC patients (245, 246).

The molecular and cellular cues underlying this delicate equilibrium between tolerance (*vis-à-vis* of self and food antigens) and immunity (against pathogens and tumors) in the intestinal barrier remain an immunological challenge.

**Figure 7:**
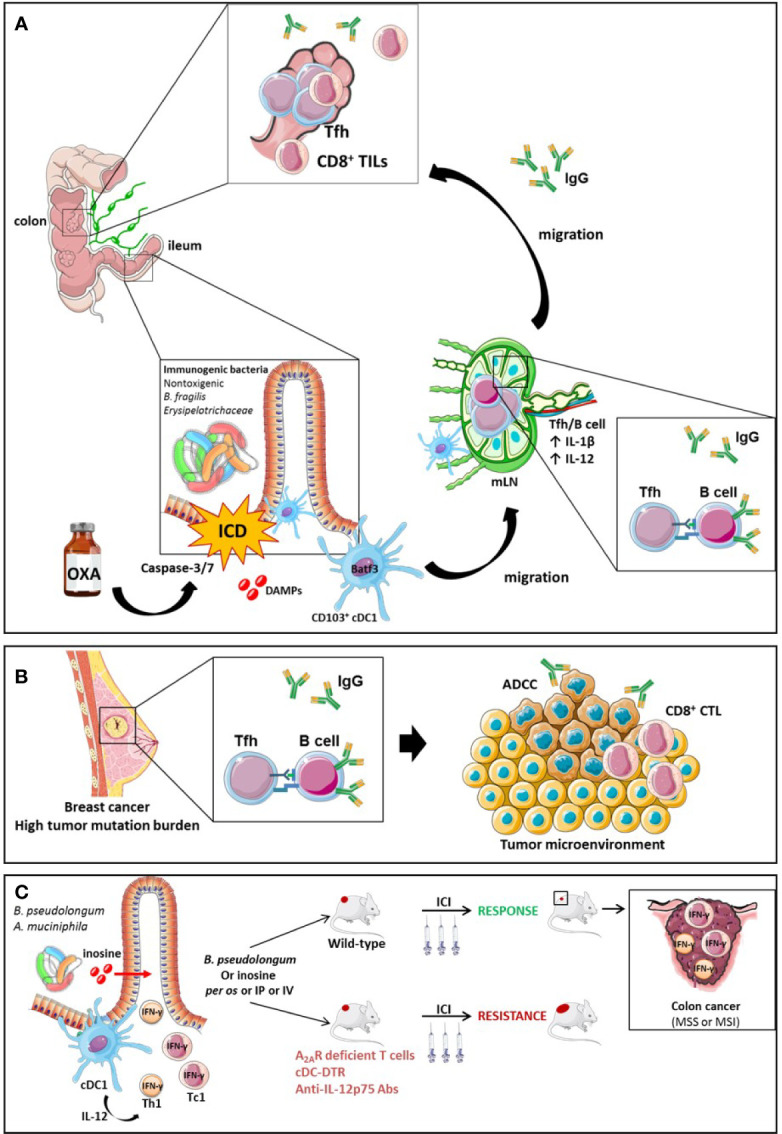
The role of Tfh and B cell orchestration for the efficacy of ICI in TMB high breast tumors or MSS CRC endowed with an immunogenic ileal microbiome. The efficacy of OXA in mouse model of CRC is driven by ileal features. On one hand, OXA induces apoptosis and the release of DAMPs following caspase-3/7-dependent ileal epithelial cell death of the crypts. The bacterial composition is critical to turn this cell death into immunogenic cell demise instead of a tolerogenic cell death. The ICD triggers the migration of CD103^+^ conventional dendritic cells (BATF3^+^ cDC1) to the mesenteric lymph node (mLN), which prime Tfh cells in an IL-1β and IL-12 dependent manner. Next, a crosstalk between Tfh and B cells occurs, leading to a systemic IgG2b response and an accumulation of Tfh and TILs into MSS or MSI colon tumors. The IgG2b responses are suspected to be directed either against bacteria or tumor cells. **(A)** Activation mechanisms between B cells and Tfh result in the generation of antibodies that elicit antibody-dependent cellular cytotoxicity (ADCC) towards tumor-associated antigens in mouse model of breast cancers endowed with a high tumor mutational burden (TMB) **(B)**. Small intestine immunogenic species (*B.pseudolongum* or *A.muciniphila*) secrete the inosine, a metabolite activating Th1 and Tc1 cells through the A_2A_. Conjointly with IL-12 co-stimulatory signal produced by cDCs in presence of antigens, inosine-stimulated Th1 and Tc1 secrete IFN-γ and increase ICIs efficacy in MSS or MSI mouse models of colon cancer **(C)**.

Recent work has highlighted how ileal bacteria may contribute to break tolerance to self-antigens shared between ileal crypts and cancer stem cells. Roberti et al. reported that the immunogenicity of ileal epithelial cell death mediated by OXA to treat a proximal CRC relied on two biological features: (1) antigenicity provided by the caspase 3/7-dependent apoptosis of crypt-derived IEC and (2) the adjuvanticity of selected ileal bacterial families or species (*Erysipelotrichaceae, B. fragilis*). These features cooperated to elicit Tfh immune responses and antibody producing cells protective against tumor progression. The serum IgG levels were increased by immunogenic bacteria but not by tolerogenic bacteria and could be associated with IgG responses directed toward bacteria or tumor cells (**Figure 7A**). A vaccine composed of OXA-exposed dying ileal IEC was more effective to immunize naive hosts against a lethal dose of colon cancer cells (CT26 and MC38) when harvested from SPF wild type mice than TLR2/4 or MYD88 KO mice or germ-free animals. These data suggest that self-antigens derived from ileal crypts could elicit an immune response in the presence of microbial adjuvants. Using 16S rRNA gene sequencing of patients’ ilea and culturomics, the authors concluded that distinct bacteria residing in ilea may convey the immunogenicity of apoptotic cell death of the crypt, shifting the antitumor immune response from Th17 (observed in progressive tumors) to Tfh cells accumulating in tumor draining lymph nodes (tdLN) of mice responding to OXA. Indeed, *Bacteroides fragilis*, or *Erysipelatoclostridium ramosum* could potentially restore responsiveness to chemotherapy in germ free mice or ATB-treated SPF animals or could confer immunogenicity to sterile ileal apoptosis. In contrast, *Fusobacterium nucleatum* or *Prevotella clara* failed to do so. In fact, OXA could mobilize migratory conventional type 1 DCs (cDC1) (CD103^+^CD11b^-^ DC) from the ileal lamina propria to the mLN, and contribute to IL-1β and IL-12-dependent priming of CXCR5^+^Bcl6^+^ PD-1^+^ CD4^+^ Tfh cells and systemic IgG responses. In the absence of Tfh or B cells, the vaccine composed of OXA-exposed crypt ileal enterocytes failed to induce long term memory autoreactive CD4^+^ and CD8^+^ T cell responses protective against colon carcinoma. In *Batf3* gene deficient animals defective in cDC1, Tfh were not primed during OXA administration. Moreover, cDC1 exposed to immunogenic ileal-residing bacteria (B. *fragilis* or *C. ramosum*) could produce IL-1β and IL-12p70 while they could not produce IL-12p70 when stimulated with *F. nucleatum* or *P. clara*. The authors postulated that PD-1 expressing Tfh induced by OXA-mediated ileal apoptosis could be bolstered by anti-PD-1 Abs and carried out to modulate the efficacy of a combination of OXA with this immune checkpoint inhibitor with a concomitant oral gavage with immunogenic (*B. fragilis, C. ramosum*) versus tolerogenic (*F. nucleatum, P. clara*) bacteria in MSS and MSI mouse colon cancers. They could bring evidence that the ileal residence and/or colonization of bacteria fostered (as for *B. fragilis, C. ramosum*) or blunted (as for *F. nucleatum, P. clara*) the immunostimulatory anticancer effects of the combinatorial regimen. These additive effects of both therapeutic modalities were accompanied by a rise in IgG2b serum levels.

Initially, ileal bacteria participate in ileal crypt apoptosis but not in the release of DAMPs. Indeed, in the presence of antibiotics, OXA-induced ileal cell demise was significantly reduced. Interestingly, OXA alone could promote the release of ATP and HMGB1, two hallmarks of ICD, that were not mandatory for the immunizing capacities of dying IEC, in contrast to live bacteria or pathogen associated molecular patterns (26). This is in contrast with a report utilizing gram negative bacteria ghosts injected intraperitoneally with OXA that could turn on ICD in the peritoneal cavity and elicit potent NKT and CD8^+^ T cell responses associated with regression of peritoneal carcinogenesis (247). These observations emphasize that the tumor macroenvironment and organ may critically influence the requirements for efficient priming of a T cell response.

Secondly, it is intriguing that ileum may be more appropriate than the colon mucosae to elicit Tfh immunity in the mLN. As outlined above (248, 249), the immune cell types and microbiota composition are highly compartmentalized (250, 251), lymphatics draining the small and large intestines differ and ileal crypts can undergo apoptotic cell death after chemotherapy while colonic crypts failed to do so (26).

Third, breaking tolerance to self-tissues in colon cancer has been previously achieved (252). The authors showed the capacity of *Stat3*-deficient IEC to promote adaptive immunity in spontaneous models of carcinogenesis (252). Mitophagy, the specific degradation process of damaged or aged mitochondria, triggered lysosomal membrane permeabilization which enhanced MHC-I expression in IEC that in turn allowed cross-dressing of DC with IEC derived-MHC-I/peptide complexes. This pathway could also come into action upon OXA treatment, knowing that STAT3 deficiency in epithelial cancer cells amplified an OXA-elicited type 1 IFN response and triggered an immunogenic cell death pattern, ameliorating antitumor T cell responses (253).

Fourth, our findings raise the theoretical possibility of a self-reactive T cell-dependent immunity that spares healthy tissues to specifically combat cancer (26). Uncoupling antitumor effects from ileitis or colitis remains an open conundrum in cancer chemotherapy and immunotherapy. We postulate that Lgr5 negative crypt-derived cells devoid of stemcellness share common antigens with pCC that elicit therapeutically relevant immune responses, at least in the context of chemotherapy with OXA. As a possibility, the fragments of apoptotic ileal IEC (that contain colon cancer-crossreactive antigens) and immunogenic commensals (with their TLR ligands) could end up in the same phagosomes of antigen-presenting cells in the GALT, thus providing an opportunity for both self-and non-self-peptides to be concomitantly loaded into MHC class II molecules (254). Actually, interactions between cytokine-producing Th cells and MHC class II^+^ Lgr5^+^ intestinal stem cells were shown to be critical for the self-renewal and/or differentiation of this latter cell type (255).

Finally, although Tfh and Tfh-like populations have been associated with poor prognosis in lymphoma (256, 257) and ICI-treated melanoma (258), numerous pre-clinical and clinical studies revealed their positive impact on the outcome of many solid tumors (259), including breast cancer (260, 261), non-small-cell lung cancer (NSCLC) (262), ovarian cancer (263), and colorectal cancer (25, 26). Mechanisms underlying the protective role of Tfh cells are not fully understood, although some evidence point to cooperation with CD8^+^ T cells and IL-21 (25, 43). CXCL13-producing Tfh act as “organizers” of germinal centers in secondary and tertiary lymphoid organs, sustaining local humoral immunity by attracting antibody-secreting B cells (264), which control tumor progression (260, 265–268). ICIs are used to reinvigorate cellular immunity and accumulating evidence show a direct impact of ICIs on humoral response. Indeed, anti-PD-1 antibodies increase germinal center formation, CD4^+^ T infiltrates and terminal B cell differentiation (269). The cooperation between Tfh and B cells appears to be associated with a favorable clinical outcome in colorectal cancer (25) and in breast cancer, in both human (260, 270) and murine models of tumors harboring high tumor mutational burden (30). The humoral response in CRC may be directed towards several tumor-associated antigens, such as Carcinoembryonic antigen (CEA) (271, 272), Epidermal Growth Factor Receptor (EGFR) (273), Human Epidermal Growth Factor Receptor 2 (HER2 or ErbB2) (274), MUC5AC (275), and ribosomal P proteins (276) and facilitate antibody-dependent cellular cytotoxicity (30) (**Figure 7B**).

Intratumoral terminally differentiated memory B cells and/or plasma cells can predict prolonged survival (49, 51, 52). Tumor-infiltrating B cells have been associated with positive clinical outcome following therapy with ICIs in melanoma (40, 277), with (41) or without metastasis (278), in sarcoma (39) and in renal cell carcinoma (40). Of note, B cells can exert direct tumoricidal activity against cancer cells, in an antigen-specific and Fas ligand-dependent manner (279).

Intratumoral bacterial colonization and residency, whether localized within myeloid cells or in tumor cells have recently been described to modulate local metabolism, change tumor cell biology or interfere in the drug catabolism (211, 280). IgG responses directed against intratumoral bacteria may contribute to preventing tumor colonization and/or tumor killing, in both mouse models and patients (30, 281, 282).

Collectively, these studies highlight the potential clinical significance of B cells, antibody secreting cells, and antibodies as well as Tfh in dictating the efficacy of the combination of FOLFOX+ICIs.

Moreover, Mager et al. have recently shown that metabolites of small intestine-derived immunogenic species, such as *Bifidobacterium pseudolongum*, can act as co-stimulatory molecules on the TCR signaling of Th1 and Tc1 cells. Indeed, *B. pseudolongum* produces the inosine, a metabolite that acts through the adenosine A_2A_ receptor expressed on TILs and potentiates the effect of PD-1 or CTLA-4 antibodies in mice bearing transplantable MC38 colon cancer in conjunction with co-stimulatory signal. This effect was lost in mice harboring A_2A_ receptor-deficient T cells. The anti-tumor effect of the inosine to reduce tumor growth is still dependent on the presence of a microbiota since in germ-free mice the administration of inosine alone did not ameliorated ICIs efficacy while it did in the presence of a complex microbiota. Additionally, *B. *pseudolongum modulated the intrinsic local immunity as it increased the T-bet expression among CD4^+^ T cells in the GALT and the mLN but not in the periphery. On the other hand, *B. pseudolongum* induced systemic immune modulation when associated with ICIs leading to the activation of effector cells and the secretion of IFN-γ by CD4^+^ and CD8^+^ T cells. The systemic effect can be explained by the fact that anti-CTLA-4 alters the gut barrier integrity increasing the systemic translocation of metabolites from the gut. The activation of T cells requires the presence of an IL-12 co-stimulatory signal provided by cDCs. Thus, in the absence of cDCs (cDC-DRT mouse model), *B. pseudolongum* associated with anti-CTLA-4 failed to induce IFN-γ-T cell producers and anti-tumoral effect. Furthermore, anti-IL-12p75 neutralization dramatically dampened the effect of anti-CTLA-4 combined with immunogenic species in a genetically modified mouse model presenting dMMR intestinal tumors mismatch repair (*Msh2^LoxP^* ^/^ *^LoxP^Villin-Cre* tumors) (**Figure 7C**) (244).

Altogether, these studies point to the central role of the ileal, but not the colonic, epithelial cells and its natural microbial ecosystem, in mobilizing an efficient immune response against self-antigens expressed by colon cancers, presumably tumor stem cells. The solution to the paradox of colon cancers-which has the propensity to elicit and attract CD3^+^/CD8^+^ T cells (so called “high immunoscore”) but remains resistant to immunotherapy when devoid of mutated neoantigen-relies on two pillars, i) ileal apoptosis through activation of the executioner caspases, and ii) a proper ileal microbiome composition balancing immunogenic and tolerogenic commensals. These two conditions can be achieved by cytotoxic regimen inducing immunogenic cell death of tumor and/or crypt stem cells and by doing so, favor the dominance of immunogenic bacteria. Such suitable therapeutic regimens include FOLFOX (5-FU/leucovorin/OXA), FOLFIRI, or any type of antibody-dependent cytotoxicity through various payloads releasing compounds endowed with ICD properties (283). The ensuing immune response is composed of humoral (Ab secreting cell and B cell-based) and cellular (Tfh and memory CD8^+^ T cell-based) based-immune responses towards crypt -derived- self antigens shared between the small intestine and tumor cells. These premises allowed the demonstration of synergistic effects between ICD-mediating chemotherapy and PD-1 blockade in pMMR or MSS mouse colon tumors.

## Conclusion

Despite tremendous advances in the classification of CRC based on genetics, transcriptomics and immunometrics, few patients benefit from combinatorial regimens that combine ICD-mediating cytotoxicants and ICIs or targeted therapies. Increasing knowledge on the macroenvironment of colon carcinoma, mainly on the connections between the local microbial ecosystem and the systemic immune tonus, has shed new light on the role of intratumoral and ileal bacteria in modulating the immune contexture as well as tumor signaling pathways. This review gives novel insights into the development of potential biomarkers of response (ileobiome, ileal immune patterns) or novel therapies based on live bio-therapeutics consisting of appropriate mixtures of immunogenic commensals or phages (284) to complement current combinatorial regimen. Moreover, attention should be paid to new actors playing a role in colon cancer immunosurveillance, including antibodies to self-antigens, B cells, and Tfh. Moreover, technological advanced such as single cell sorting and sequencing will also enable a comprehensive characterization of these actors’ functions and specificities.

## Author’s Note

The figures were conceived using https://smart.servier.com/.

## Author Contributions

MF, SY, MP, AH, AC, MR, and LZ contributed to the writing of this manuscript. MF and MR conceived the figures using https://smart.servier.com/. All authors contributed to the article and approved the submitted version.

## Funding

LZ was supported by the Ligue contre le Cancer (équipe labelisée); Association pour la recherche sur le cancer (ARC); Agence Nationale de la Recherche (ANR) francogermanique ANR-19-CE15-0029, Cancéropôle Ile-de-France; Fondation pour la Recherche Médicale (FRM); Institut National du Cancer (INCa); Inserm (HTE); the LabEx Immuno-Oncology; the RHU Torino Lumière (ANR-16-RHUS-0008); H2020 ONCOBIOME, the Seerave Foundation; the SIRIC Stratified Oncology Cell DNA Repair and Tumor Immune Elimination (SOCRATE); FHU CARE, Dassault, and Badinter Philantropia, and the Paris Alliance of Cancer Research Institutes (PACRI). MF was supported by AP-HP. MP was supported by H2020 ONCOBIOME. SY was supported by Fondation Philanthropia, Gustave Roussy. AC is supported by the France Fulbright Comission and the Cancer Research and Prevention Insitute of Texas (CPRIT), Research Training Program (RP170067).

## Conflict of Interest

The authors declare that the research was conducted in the absence of any commercial or financial relationships that could be construed as a potential conflict of interest.

## Abbreviations

5-FU, 5-fluorouracil; Ab, antibody; ADCC, antibody-dependent cellular cytotoxicity; ATB, antibiotics; Casp, caspase; cDC, conventional dendritic cell; CDD_L_, enzyme cytidine deaminase; CIMP, CpG island methylator phenotype; CIN, chromosomal instability; CMS, consensus molecular subtype; CPT-11, irinotecan; CRC, colorectal cancer; CTL, cytotoxic T lymphocyte; DAMP, danger associated molecular pattern; d/pMMR, deficient/proficient DNA mismatch repair; EMT, epithelial-mesenchymal transition; ETBF, enterotoxigenic *Bacteroides fragilis*; FMT, fecal microbial transplantation; GALT, gut-associated lymphoid tissues; GF, germ free; GI, gastrointestinal; HMGB1, high mobility group box 1 protein; I.S., immune subtypes; IS, immunoscore; ICD, immunogenic cell death; ICI, immune checkpoint inhibitors, IEC, intestinal epithelial cells; KEGG, Kyoto Encyclopedia of Genes and Genomes; KO, knock-out; LP, *Lamina Propria*; MDSC, myeloid-derived suppressor cell; MGS, metagenomic shotgun; mLN, mesenteric lymph node; MSI/MSS, microsatellite instability/stability; NK, natural killer; NSCLC, non-small lung cancer; NTBF, non-toxigenic *Bacteroides fragilis*; OS, overall survival; OXA, oxaliplatin; pCC, proximal colon adenocarcinoma; pDC, plasmacytoid dendritic cell; PFS, progression-free survival; ROS, reactive oxygen species; SCFA, short-chain fatty acid; SPF, specific pathogen free; Tc1, T cytotoxic type 1; TCGA, The Cancer Genome Atlas; tdLN, tumor draining lymph node; TFC, T follicular cell; Tfh, T follicular helper cell; Th, T helper; TILs, tumor-infiltrating lymphocytes; TLR, Toll-like receptor; TMB, tumor mutational burden; TME, tumor microenvironment; TNM, T-primary tumor, N-regional lymph nodes, M-distant metastases; Treg, T regulatory cell; WT, wild type.
